# Supplementary material for: Estimating Effect Sizes and Expected Replication Probabilities from GWAS Summary Statistics
Source: Front Genet. 2016 Feb 16;7:15. doi: 10.3389/fgene.2016.00015 (PMC4754432; doi:10.3389/fgene.2016.00015)
Supplement: Supplementary file 1 [file Presentation1.pdf]

Supporting Material: Estimating Effect Sizes and Expected Replication Rates from GWAS Summary Statistics

Dominic Holland[a,b,*], Yunpeng Wang[a,b,c,d], Wesley K. Thompson[e], Andrew Schork[a,f], Chi-Hua Chen[a,g], Min-Tzu Lo[a,g], Aree Witoelar[c,d], Schizophrenia Working Group of the Psychiatric Genomics Consortium, Enhancing Neuro Imaging Genetics through Meta Analysis Consortium, Thomas Werge[h], Michael O'Donovan[i], Ole A. Andreassen[c,d], Anders M. Dale[a,b,e,g]

[a]*Multimodal Imaging Laboratory, University of California at San Diego, La Jolla, CA 92093, USA,*
[b]*Department of Neurosciences, University of California, San Diego, La Jolla, CA 92093, USA,*
[c]*NORMENT, KG Jebsen Centre for Psychosis Research, Institute of Clinical Medicine, University of Oslo 0424 Oslo, Norway,*
[d]*Division of Mental Health and Addiction, Oslo University Hospital, 0407 Oslo, Norway,*
[e]*Department of Psychiatry, University of California, San Diego, La Jolla, CA 92093, USA,*
[f]*Department of Cognitive Sciences, University of California at San Diego, La Jolla, CA 92093, USA,*
[g]*Department of Radiology, University of California, San Diego, La Jolla, CA 92093, USA,*
[h]*Institute of Biological Psychiatry, MHC, Sct. Hans Hospital and University of Copenhagen, 4000 Copenhagen, Denmark,*
[i]*MRC Centre for Neuropsychiatric Genetics and Genomics, School of Medicine, Cardiff University, Heath Park, Cardiff, CF14 4XN, UK,*

METHODS

Basic Linear Model

In order to characterize the distribution of effect sizes that single nucleotide polymorphisms (SNPs) have on a particular phenotype, consider the usual linear regression framework using the additive genetic model:

$$Y = G\beta + e. \tag{1}$$

Here, Y is a vector of phenotypic values for the N subjects in the study, and G is the $N \times n$ genotype matrix for their n SNPs: $g_{ij} = 0$, 1, or 2, the number of reference alleles at the jth SNP for the ith subject; e is the residual effect, an N-vector of random errors and environmental contributions, assumed to be exogenous (zero-centered and uncorrelated with the regressors: $E(e|G) = 0$, where E denotes expectation). The data elements $\{y_i, G_i\}$, where G_i is the genotype n-vector (over SNPs) for the ith subject, are considered to be random and sampled from a population. This means that β, the vector of regression coefficients, is an n-vector of random genetic effects (a source of variance, in addition to e), whose means and variances are of primary interest. Thus, all three quantities on the right-hand side of Eq. 1 are considered random. Let $R_e = \mathrm{var}(e)$, the covariance matrix for e, and $R_\beta = \mathrm{var}(\beta)$, the covariance matrix for β; denote transpose with a prime, and let $\hat{}$ denote estimates of true values from the data. Then the best linear unbiased predictor (BLUP) (Robinson, 1991) $\hat\beta$ for β is

$$\begin{aligned}\hat\beta &= (G'R_e^{-1}G + R_\beta)^{-1}G'R_e^{-1}Y \\ &= R_\beta G'(R_e + GR_\beta G')^{-1}Y \\ &\equiv WY \end{aligned} \tag{2}$$

with

$$\widehat{\mathrm{var}}(\hat\beta) = W(GR_\beta G' + R_e)W'. \tag{3}$$

The first thing to do is to simplify. (1) Assume the residuals are uncorrelated and have constant variance: $R_e = \sigma_e^2 I_N$, where I_N is the $N \times N$ identity matrix and σ_e is a constant. (2) Assume the random effects β are uncorrelated and that, in a Bayesian sense, the priors on them are all equally and highly uninformative (they have very large variance): $R_\beta = \sigma_\beta^2 I$, with $\sigma_\beta \gg \sigma_e$. Eqs. 2 and 3 then reduce to the familiar ordinary least squares (OLS) solution to Eq. 1 (minimizing only the error variance, $\hat\sigma_e^2$)

$$\hat\beta = (G'G)^{-1}G'Y \tag{4}$$

with

$$\widehat{\mathrm{var}}(\hat\beta) = \hat\sigma_e^2(G'G)^{-1}, \tag{5}$$

and $\hat\sigma_e^2 = \mathrm{var}(Y - \widehat{Y})$ where $\widehat{Y} = G\hat\beta$. With more regression coefficients than data points, $n \gg N$ (the usual situation in GWAS), there is no unique solution for β. A further simplification is to resort to simple linear regression, treating each SNP independently (the massively univariate approach, ignoring correlation between SNP genotypes): only the diagonal of $G'G$ in Eqs. 4 and 5 is considered to be non-zero. Essentially, for each SNP j in turn (j=1,...,n; no summation over repeated indices), solve the set of N equations

$$y_i = g_{ij}\beta_j + \varepsilon_{ij} \qquad i = 1, ..., N, \tag{6}$$

where i indexes the subjects and ε_{ij} is the residual. In a case-control situation ($y_i = 1$ for cases and $y_i = 0$ for controls), one performs logistic regression, replacing the left-hand side of Eq. 6 with

$$\theta_i = \mathrm{logit}[Pr(y_i = 1|g_{ij})],$$

where $Pr(y_i = 1|g_{ij}) = E(y_i = 1|g_{ij})$, with Pr denoting probability. To relate estimated quantities on the observed

*Corresponding author:
email: dominic.holland@gmail.com
Phone: 858-822-1776
Fax: 858-534-1078

case-control (0/1) scale to an underlying continuous liability scale, and taking into consideration case-control ascertainment and disease prevalence, see Lee et al. (2012).

From Eq. 6, the estimate of the element $\hat{\beta}_j$ is given by the corresponding diagonal entry from the OLS estimate

$$\hat{\beta}_j = G_j' Y / (G_j' G_j) \tag{7}$$

where now G_j denotes the N-vector (over subjects) of genotype values for the jth SNP, and

$$\widehat{\mathrm{var}}(\hat{\beta}_j) = \hat{\sigma}^2_{\varepsilon,j} (G_j' G_j)^{-1}, \tag{8}$$

with

$$\hat{\sigma}^2_{\varepsilon,j} = \frac{1}{N-2} \sum_{i=1}^{N} \hat{\varepsilon}^2_{ij}$$

where

$$\hat{\varepsilon}_{ij} = y_i - g_{ij}\hat{\beta}_j$$

(again, no summation over repeated indices). For a polygenic phenotype each SNP will contribute only a small amount to the phenotypic variance. Therefore all the $\hat{\sigma}^2_{\varepsilon,j}$ will be approximately equal to (but slightly less than) the phenotypic variance: $\hat{\sigma}^2_{\varepsilon,j} \simeq \widehat{\mathrm{var}}(y)$.

If G is centered (for each SNP, the mean value over subjects is subtracted from the integral genotypic value for each subject), $G_j = g_j - \bar{g}_j$, $g_j = (g_{1j}, ..., g_{ij}, ...g_{nj})$, and letting $\mathrm{var}(g_j)$ denote the scalar variance arising from the vector elements, then

$$\begin{aligned} G_j' G_j &= \sum_{i=1}^{N} (g_{ij} - \bar{g}_j)'(g_{ij} - \bar{g}_j) & (9)\\ &= N \mathrm{var}(g_j), & (10) \end{aligned}$$

a result that holds regardless of the centering (since for $a \in \mathbb{R}$, $\mathrm{var}(a + g_i) = \mathrm{var}(g_i) = \mathrm{var}(G_i)$), and $\hat{\beta}$ can be written as $\hat{\beta} = \mathrm{cov}(G_j, Y)/\mathrm{var}(G_j)$. Under Hardy-Weinberg equilibrium, g_{ij} is distributed binomially with allele frequency p_j: $g_{ij} \sim \mathrm{Bin}(2, p_j)$. Then

$$\begin{aligned} G_j' G_j &= N 2 p_j (1 - p_j) \\ &\equiv N H_j & (11) \end{aligned}$$

where H_j is the heterozygosity (allele count variance) for SNP j. Therefore, one can write

$$\widehat{\mathrm{var}}(\hat{\beta}_j) = \frac{\hat{\sigma}^2_{\varepsilon,j}}{N H_j} \equiv [\widehat{\mathrm{se}}(\hat{\beta}_j)]^2, \tag{12}$$

se denoting standard error.

Basic Gaussian Model

The Wald statistic corresponding to $\hat{\beta}_j$ is given by

$$\begin{aligned} z_j &= \frac{\hat{\beta}_j}{\widehat{\mathrm{se}}(\hat{\beta}_j)} \\ &= \frac{\hat{\beta}_j \sqrt{N \cdot H_j}}{\hat{\sigma}_{\varepsilon,j}}. & (13) \end{aligned}$$

Dropping the subscript j indicating a particular SNP, one can write $\hat{\beta} = E(\hat{\beta}) + \gamma$, where γ is an environment and error term with $E(\gamma) = 0$. Note that OLS (and simple linear regression) estimates are unbiased estimates of β. We assume $E(\hat{\beta}) \simeq \beta$. Then

$$\begin{aligned} z &= \frac{\sqrt{N \cdot H} \cdot \beta}{\sigma_\varepsilon} + \frac{\sqrt{N \cdot H} \cdot \gamma}{\sigma_\varepsilon} \\ &\equiv \delta + \epsilon & (14) \end{aligned}$$

where δ is the genetic effect size and ϵ is due to environment and error and assumed to be independent of δ (thus ignoring any genotype-phenotype interactions). Under the null hypothesis of no effect, $\beta = 0$ and z is assumed to be normally distributed with mean 0 and variance σ_0^2 (which might be slightly different from 1 due to population structure (Devlin and Roeder, 1999)): $z \sim \mathcal{N}(0, \sigma_0^2)$. Thus,

$$\delta \propto \sqrt{N \cdot H} \cdot \beta, \tag{15}$$

and

$$\begin{aligned} \mathrm{var}(z) &= \mathrm{var}(\delta) + \mathrm{var}(\epsilon) \\ &\equiv \sigma_1^2 + \sigma_0^2 & (16) \end{aligned}$$

where

$$\sigma_1^2 \equiv \sigma_a^2 N \cdot H \tag{17}$$

with σ_a^2, the constant of proportionality (independent of the quantities N and H), being the per-allele variance – the variance component associated with each allele. Motivated by the central limit theorem, the essential component of the basic Gaussian model is to assume that δ has a normal prior distribution with mean 0 and variance σ_1^2: $\delta \sim \mathcal{N}(0, \sigma_1^2)$.

In summary, the usual single group Gaussian model is:

$$\begin{aligned} & z = \delta + \epsilon, & \delta \text{ and } \epsilon \text{ indep.} \quad (18)\\ & \epsilon \sim \mathcal{N}(0, \sigma_0^2), & (19)\\ & \delta \sim \mathcal{N}(0, \sigma_1^2), & \text{prior density of } \delta \quad (20)\\ & z|\delta \sim \mathcal{N}(\delta, \sigma_0^2), & \text{likelihood of } \delta \text{ given } z. \quad (21) \end{aligned}$$

Then, using the familiar property of Gaussian integrals,

$$\int_{-\infty}^{+\infty} e^{-(ax^2+bx)}\,\mathrm{d}x = \sqrt{\frac{\pi}{a}} e^{b^2/(4a)}, \tag{22}$$

and Bayes equation, $Pr(\delta|z) = Pr(z|\delta)Pr(\delta)/Pr(z)$, where the marginal distribution of z-scores is given by the convolution

$$\begin{aligned} f(z) \equiv Pr(z) &= \int Pr(z|\delta) Pr(\delta) \mathrm{d}\delta \\ &= \int \phi(z; \delta, \sigma_0^2) \phi(\delta; 0, \sigma_1^2) \mathrm{d}\delta \\ &= \int \phi(z - \delta; 0, \sigma_0^2) \phi(\delta; 0, \sigma_1^2) \mathrm{d}\delta \\ &= \phi(z; 0, \sigma_0^2 + \sigma_1^2), \end{aligned}$$

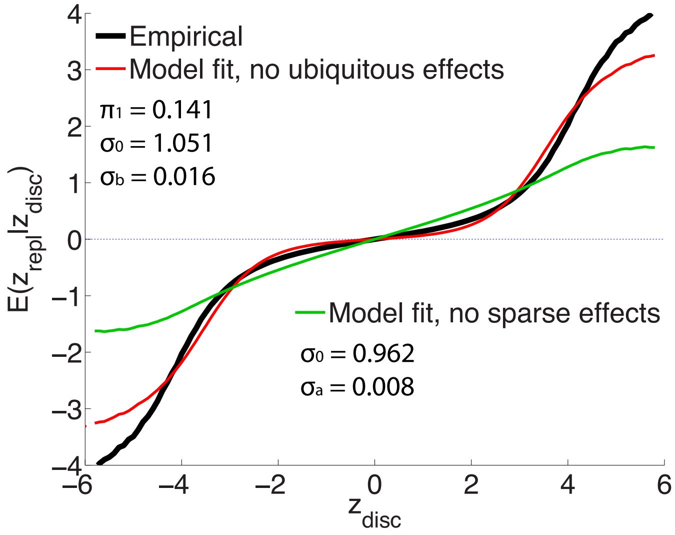

Figure 1: Posterior estimates of effect size for schizophrenia, comparing the empirical estimates with those from explicit model fits with (a) three parameters for no ubiquitous component (no σ_a, red curve), and (2) two parameters for no sparse component (no σ_b, green curve). In the latter case, note that the model is degenerate with respect to π_1. Compare with Fig. 1(A) in the main paper.

where $\phi(\cdot;\mu,\sigma^2)$ is the normal PDF with mean μ and variance σ^2, one has

$$\delta|z \sim \mathcal{N}(Az, A\sigma_0^2), \qquad \text{posterior distb. of } \delta \text{ given } z \quad (23)$$

$$A \equiv \frac{\sigma_1^2}{\sigma_0^2+\sigma_1^2}, \quad (24)$$

$$z \sim \mathcal{N}(0, \sigma_0^2+\sigma_1^2), \qquad \text{marginal distb. of } z. \quad (25)$$

Thus, in the case of the single-group (non-mixture) model (Eq. 25),

$$E(\delta|z) = z\frac{\sigma_1^2}{\sigma_0^2+\sigma_1^2} \equiv \mu_u(z,H,N), \quad (26)$$

$$\text{var}(\delta|z) = \sigma_0^2\frac{\sigma_1^2}{\sigma_0^2+\sigma_1^2} \equiv \sigma_u^2(H,N). \quad (27)$$

Noting that $\delta \propto \sqrt{N}$, then for a z-score measured in a sample of effective sample size N_d the expected posterior effect size δ_r in a new sample of effective sample size N_r is given by

$$E(\frac{\delta_r}{\sqrt{N_r}}|z_d) = E(\frac{\delta_d}{\sqrt{N_d}}|z_d). \quad (28)$$

That is,

$$E(\delta_r|z_d) = \sqrt{\frac{N_r}{N_d}}\mu_u. \quad (29)$$

Similarly,

$$\text{var}(\delta_r|z_d) = \frac{N_r}{N_d}\sigma_u^2. \quad (30)$$

For probability densities that are more intractable than simple Gaussians, the standard formalism of a one parameter exponential family of probability densities can be used to estimate means and variances (Efron, 2013). As an illustration, Eq. 23 can be re-expressed in terms of an exponential family as

$$Pr(\delta|z) = [Pr(\delta)e^{-\delta^2/(2\sigma_0^2)}]e^{(\delta/\sigma_0^2)z}e^{-\psi(z)}, \quad (31)$$

where

$$\psi(z) \equiv \ln\left(\frac{f(z)}{\phi(z,0,\sigma_0^2)}\right), \quad (32)$$

with $Pr(\delta)$ given by Eq. 20, and $f(z)$ given by Eq. 25. Writing $T(\delta) \equiv \delta/\sigma_0^2$, one has from the exponential family formalism

$$E[T(\delta)|z] = \psi'(z) \quad (33)$$

and

$$\text{var}[T(\delta)|z] = \psi''(z). \quad (34)$$

Letting $l(z) \equiv ln[f(z)]$, one has immediately

$$E(\delta|z) = z + \sigma_0^2 l'(z) \quad (35)$$

and

$$\text{var}(\delta|z) = \sigma_0^2[1+\sigma_0^2 l''(z)], \quad (36)$$

from which Eqs. 26 and 27 follow directly.

Gaussian Mixture Model

The motivation for pursuing a two-groups mixture model, as discussed in the main text, comes from the empirical results (black curve in Fig. 1) which indicate that the z-scores show evidence of having both ubiquitous ("small") effects and sparse ("large") effects: small but non-zero slope around the origin, transitioning to steep slope further away from the origin. A single-group distribution like Eq. 25 does not well characterize the data, as shown by the green curve in Fig. 1, and even a two-groups mixture model that does not incorporate ubiquitous effects provides only an inaccurate description, as shown by the red curve in Fig. 1.

The two-groups mixture model, that is an extension of Eq. 25 proposed here, is given by Eq. 1 in the main paper:

$$f(z) = \pi_0\phi(z,0,\sigma_0^2+\sigma_1^2) + \pi_1\phi(z,0,\sigma_0^2+\sigma_1^2+\sigma_2^2), \quad (37)$$

i.e.,

$$z \sim \pi_0\mathcal{N}(0,\sigma_0^2+\sigma_1^2) + \pi_1\mathcal{N}(0,\sigma_0^2+\sigma_1^2+\sigma_2^2) \quad (38)$$

(a mixture of scaled Gaussians) where, analogous to Eq. 17,

$$\sigma_2^2 \equiv \sigma_b^2 N \cdot H \quad (39)$$

σ_b^2 being the additional variance component associated with alleles exhibiting sparse effects (the additional per-allele variance for sparse effects). The empirical PDF for z-scores and the model estimate, Eq. 37 (where the model parameters were estimated as described in the main paper), are plotted in Fig. 2 for five allele count variance windows. The excellent match between the empirical and

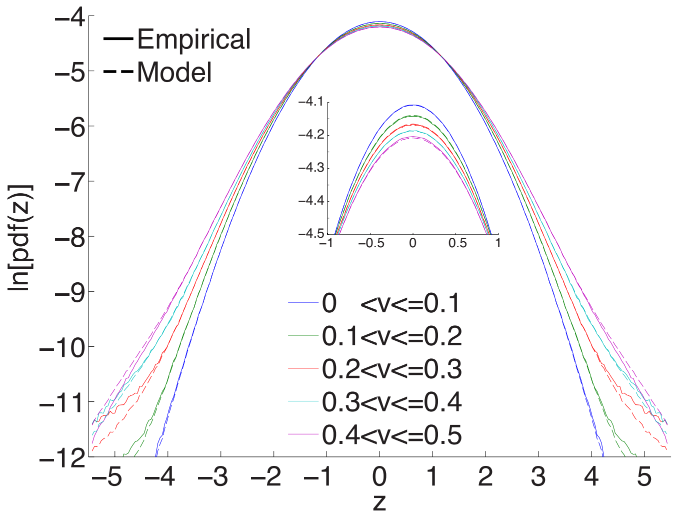

Figure 2: Empirical and model estimates of the probability density of z-scores for schizophrenia, for 5 heterozygosity windows $H = 2p(1-p)$, where p is the minor allele frequency. The model estimates are given by Eq. 37. The natural logarithm of the PDF is plotted to emphasize the tail behavior.

model plots over a wide range of z-scores indicates the validity of the model definition. In summary, one has

$$z = \delta + \epsilon, \qquad \delta \text{ and } \epsilon \text{ indep.} \tag{40}$$

$$\epsilon \sim \mathcal{N}(0, \sigma_0^2), \tag{41}$$

$$\delta = \begin{cases} \delta_a & \text{with prior prob. } \pi_0 \\ \delta_a + \delta_b & \text{with prior prob. } \pi_1 \end{cases} \tag{42}$$

$$\delta_a \sim \mathcal{N}(0, \sigma_1^2), \qquad \text{prior density of } \delta_a \tag{43}$$

$$\delta_b \sim \mathcal{N}(0, \sigma_2^2), \qquad \text{prior density of } \delta_b \tag{44}$$

with δ_a and δ_b independent. As before, $z|\delta \sim \mathcal{N}(\delta, \sigma_0^2)$, but now the prior density for δ, $Pr(\delta)$, is

$$Pr(\delta) = \pi_0 \phi(\delta, 0, \sigma_1^2) + \pi_1 \phi(\delta, 0, \sigma_1^2 + \sigma_2^2). \tag{45}$$

When $\sigma_1 = 0$, the normal distribution in the first term becomes a Dirac delta function, $\Delta(\delta)$ (a discrete "atom" or point mass or point normal distribution at $\delta = 0$). In the special case where the prior probability that SNPs are associated with sparse effects approaches 1, i.e., $\pi_1 \to 1$, then the distinction between σ_1^2 and σ_2^2 becomes meaningless (only their sum matters), one has

$$E(\delta|z) = z \frac{\sigma_1^2 + \sigma_2^2}{\sigma_0^2 + \sigma_1^2 + \sigma_2^2} \equiv \mu_s, \tag{46}$$

$$\text{var}(\delta|z) = \sigma_0^2 \frac{\sigma_1^2 + \sigma_2^2}{\sigma_0^2 + \sigma_1^2 + \sigma_2^2} \equiv \sigma_s^2. \tag{47}$$

Given a z-score, the posterior probability of it belonging to the "sparse" (π_1) arm of Eq. 37 is the "true discovery rate", $tdr(z)$ (Efron, 2013), and is given by Bayes rule:

$$tdr(z, H, N) = \frac{\pi_1 \phi(z, 0, \sigma_0^2 + \sigma_1^2 + \sigma_2^2)}{f(z)}, \tag{48}$$

(if the SNP is declared to have a sparse effect, this is the probability of being correct; the false discovery rate $fdr = 1 - tdr = \pi_0 \phi(z, 0, \sigma_0^2 + \sigma_1^2)/f(z)$). Since δ and ϵ are assumed to be independent, note that $\text{var}(z_r|z_d) = \text{var}(\delta_r|z_d) + \sigma_0^2$. Thus, combining Eqs. 26, 27, 37-48, and dropping explicit dependence on $z, H,$ and N to simplify the notation, the posterior distribution of δ given z is

$$Pr(\delta|z) = (1 - tdr)\phi(\delta, \mu_u, \sigma_u^2) + tdr \cdot \phi(\delta, \mu_s, \sigma_s^2). \tag{49}$$

Additionally, the posterior distribution of z-scores z_r in a new "replication" sample of effective sample size N_r, given z_d in a "discovery" sample of effective sample size N_d, is

$$Pr(z_r|z_d) = (1 - tdr)\phi(z_r, m_u, s_u^2) + tdr \cdot \phi(z_r, m_s, s_s^2), \tag{50}$$

where

$$m_u = \sqrt{N_r/N_d} \cdot \mu_u \tag{51}$$

$$m_s = \sqrt{N_r/N_d} \cdot \mu_s \tag{52}$$

$$s_u^2 = \sigma_0^2 + (N_r/N_d) \cdot \sigma_u^2 \tag{53}$$

$$s_s^2 = \sigma_0^2 + (N_r/N_d) \cdot \sigma_s^2. \tag{54}$$

From Eq. 50, one immediately has the posterior expectation of effect size in a replication sample, give a z-score in the discovery sample:

$$E(\delta_r|z_d) = \sqrt{N_r/N_d}[(1 - tdr)\mu_u + tdr \cdot \mu_s], \tag{55}$$

which also immediately follows from Eq. 35.

Since variance is given by the second moment minus the square of the first moment, the variance of δ given z, $\text{var}(\delta|z)$, can be calculated straightforwardly from Eq. 49, which itself is in the form of a mixture of two Gaussians (with constant weights $1-tdr$ and tdr for given $z, H,$ and$N$). Thus, for fixed z, write

$$g(\delta) \equiv Pr(\delta|z) \equiv (1 - tdr)g_u(\delta) + tdr \cdot g_s(\delta), \tag{56}$$

with $g_u(\delta) \equiv \phi(\delta, \mu_u, \sigma_u^2)$, and similarly for g_s. Let q_k denote the kth moment of δ with respect to the mixture distribution g:

$$q_k \equiv E_g(\delta^k) = (1 - tdr)E_u(\delta^k) + tdr \cdot E_s(\delta^k), \tag{57}$$

with E_g denoting expectation with respect to g, and E_u and E_s denoting expectation with respect to g_u and g_s, respectively. Then,

$$q_1 = (1 - tdr)\mu_u + tdr \cdot \mu_s \tag{58}$$

and (for the individual mixture terms, recalling that the second moment is the square of the first moment plus the variance)

$$q_2 = (1 - tdr)(\mu_u^2 + \sigma_u^2) + tdr \cdot (\mu_s^2 + \sigma_s^2). \tag{59}$$

Since $\text{var}(\delta|z) = q_2 - q_1^2$, it follows that

$$\begin{aligned}\text{var}(\delta|z) &= (1-tdr)(\mu_u^2+\sigma_u^2) + tdr\cdot(\mu_s^2+\sigma_s^2) - \\ &\quad [(1-tdr)\mu_u + tdr\cdot\mu_s]^2 \\ &= (1-tdr)\sigma_u^2 + tdr\cdot\sigma_s^2 + \\ &\quad tdr(1-tdr)[\mu_s-\mu_u]^2, \qquad (60)\end{aligned}$$

which also immediately follows from Eq. 36.

Effective Sample Size

Sample size explicitly enters Wald statistics or z-scores through the denominator term, $\widehat{\text{se}}(\hat\beta) \propto \sqrt{N}$, where N is the effective sample size. For binary qualitative traits (e.g., disease / no disease), z-scores calculated through logistic regression are equivalent to those calculated using the Cochran-Arimtage trend test (Wellek and Ziegler, 2012), where one is explicitly interested in testing the null hypothesis that the allele frequencies in the affecteds (p_A) and unaffecteds (p_U) are equal, $\text{H}_0: p_A = p_U$:

$$z_T = \frac{\hat p_A - \hat p_U}{\sqrt{var(\hat p_A - \hat p_U)}}.$$

Equivalently, let X be a scaled (0,1,2) random variable giving the number of alleles of interest, and compare the means in affecteds and unaffecteds using the test statistic

$$z_T = \frac{\overline{X}_A - \overline{X}_U}{\sqrt{\text{var}(\overline{X}_A - \overline{X}_U)}}.$$

Under H_0, assume X_A and X_U have the same underlying variance, $\sigma^2 = \text{var}(X)$, and mean, μ. If there are N_A affecteds, then under H_0, $\overline{X}_A$ is normally distributed with mean μ and variance σ^2/N_A. Similarly, if there are N_U unaffecteds, then under H_0, $\overline{X}_U$ is normally distributed with mean μ and variance σ^2/N_U. Therefore, because the two samples are independent, under H_0, $(\overline{X}_A - \overline{X}_U)$ is normally distributed with mean 0 and variance

$$\sigma^2(1/N_A + 1/N_U) \equiv \sigma^2/(N_{eff}/2), \qquad (61)$$

where the effective sample size, N_{eff}, is defined as

$$N_{eff} = 2/(1/N_A + 1/N_U). \qquad (62)$$

With this definition of N_{eff}, if there are equal numbers of affecteds and unaffecteds, $N_{eff} = N_A = N_U$; i.e., N_{eff} is the number of subjects per arm of a balanced-design study. The factor 2 in Eqs. 61 and 62 does not affect the calculation of the meta-analysis z in Eq. 65 below. In the model PDF, Eq. 37, N_{eff} appears only as a multiplicative factor for the "per allele" (or per subject, with N_{eff} subjects) variance components σ_a^2 and σ_b^2. Thus, for example, if N_{eff} is redefined to be twice or half its value above (replacing 2 with 4 or 1), σ_a^2 and σ_b^2 will simply halve or double, retaining their "per allele" meaning. With N_{eff} defined as in Eq. 62, for balanced designs the total sample size for a qualitative trait is $2N_{eff}$; total sample sizes for quantitative and qualitative traits can then be directly compared.

Thus, for binary qualitative traits, given K sub-studies, for the k-th sub-study with N_A affecteds and N_U unaffecteds, the effective sample size N_k was defined as

$$N_k = \frac{2}{(1/N_A + 1/N_U)}. \qquad (63)$$

The total effective sample size over K sub-studies was then

$$N = \sum_{k=1}^{K} N_k. \qquad (64)$$

For quantitative traits, the effective sample size over K sub-studies is just the sample size given by the sum of the subjects in each sub-study.

Meta Analysis z-scores

For a given SNP, z-scores z_k, $k = 1, \ldots K$, from K sub-studies with, respectively, effective sample sizes N_k, were combined in a weighted Z-test (Liptak, 1958) to estimate the meta-analysis z-score for the SNP. Under the null hypothesis H_0 (and ignoring inflation), $z_k \sim \mathcal{N}(0,1)$ for each k. Writing the weighted z in the form

$$\begin{aligned} z &= \frac{\sum_{k=1}^K w_k z_k}{\sqrt{\sum_{k=1}^K w_k^2}} \\ &\equiv \sum_{k=1}^K c_k z_k \end{aligned}$$

for weights w_k ensures that under H_0: $z \sim \mathcal{N}(0,1)$ ($\sum_{k=1}^K c_k^2 = 1$). Following Liptak (Liptak, 1958; de Bakker et al., 2008; Zaykin, 2011), we chose the weights to be the square root of the sample size $w_k = \sqrt{N_k}$. When the substudies are from similar populations, these are optimal weights (Zaykin, 2011), and the test is equivalent to weighting by the inverse standard deviation of the statistic used in the k-th sample:

$$z = \frac{\sum_{k=1}^K \sqrt{N_k} z_k}{\sqrt{\sum_{k=1}^K N_k}}. \qquad (65)$$

(Inverse variance weighting is equivalent to choosing $w_k = N_k$, in which case the test is called the Mosteller-Bush test (Mosteller and Bush, 1954).)

Confidence Intervals for the Model Parameters

To estimate the 95% confidence intervals of the four model parameters, $K = 20$ pair estimates of the parameters were made from K split-half random divisions of the SNPs. Because the three model standard deviations (σ_0, σ_a, and σ_b) are constrained to be positive, and the polygenicity (π_1) is constrained to lie between 0 and 1, the parameters were first transformed to unconstrained forms (ranging from $-\infty$ to $+\infty$). Specifically, each standard deviation σ was estimated as $\sigma' = \ln(\sigma)$, and the polygenicity was estimated as $\pi_1' = \text{logit}(\pi_1) = \ln(\pi_1/(1-\pi_1))$. K pairs of these were then estimated with the minimization

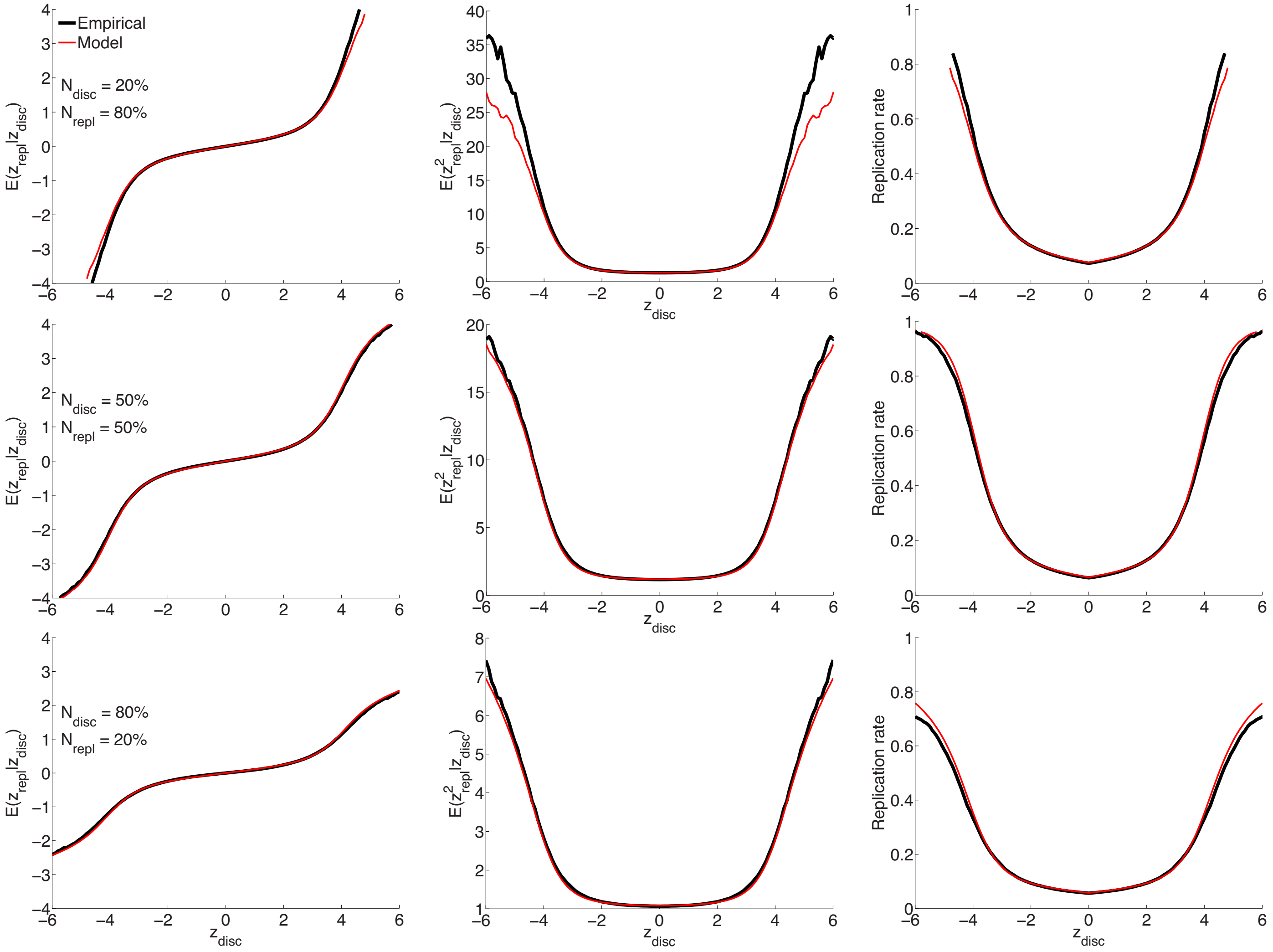

Figure 3: For schizophrenia, posterior estimate of (A) effect size and (B) square of effect size; (C) estimate of replication rate: empirical (black solid lines), current model (red solid lines), 20%, 50%, and 80% of full data as discovery sample (the complement as replication sample).

scheme described in the main paper. Split-half random divisions of the SNPs were generated by dividing the entire set of SNPs into 1000 non-overlapping blocks (the number of SNPs per block then well-exceeded the average LD block size; using a threshold of $r^2 \geq 0.1$ for the squared correlation, the number of independent SNPs was 339,161 out of a total of 9,279,485, giving on average less than 30 SNPs per LD block). Half of the blocks were randomly chosen to compose the data set for estimation of one set of the model parameters; the remaining blocks then provided an independent data set from which also to estimate the parameters. $K = 20$ such complementary random divisions were made. Note that there is data overlap, and therefore correlation, between different pairs of divisions, while elements of a pair division are independent. The variance of the difference between the pair of estimates from complementary independent data sets then provided an estimate for the variance of the corresponding model parameter, from which the standard error immediately follows. Assuming the transformed estimates are distributed multinormally, the 95% confidence interval for a (transformed) parameter was given by the point estimate from the full data set $\pm 1.96\times$ the corresponding standard error; these limits were then inverse transformed to the native parameter representation.

Let A and B be random variables for a pair of independent estimates of, say, π'_1, with realizations a and b; since only half the data were used in each of the independent estimates, one has $\mathrm{var}(A) = \mathrm{var}(B) = 2\mathrm{var}(\pi'_1)$. Then,

$$\mathrm{var}(A - B) = \mathrm{var}(A) + \mathrm{var}(B) = 2\mathrm{var}(A) = \\ E[(A - B)^2] - [E(A - B)]^2 = E[(A - B)^2].$$

$\mathrm{var}(\pi'_1)$ can be estimated from the K pairs of parameter point estimates as

$$\widehat{var}(\hat{\pi}'_1) = \frac{\widehat{E}[(A-B)^2]/2}{2},$$

and the standard error, being the standard deviation of the test statistic, is then simply

$$\widehat{se}(\hat{\pi}'_1) = \frac{1}{2}\sqrt{\frac{\sum_{i=1}^{K}[(a_i - b_i)^2]}{K}}.$$

Letting $\hat{\pi}'_1$ denote the point estimate from the entire set of SNPs of the (transformed) polygenicity parameter, the (transformed) 95% confidence interval CI' is

$$CI' = [\hat{\pi}'_1 - 1.96 \times \widehat{se}(\hat{\pi}'_1); \quad \hat{\pi}'_1 + 1.96 \times \widehat{se}(\hat{\pi}'_1)]. \quad (66)$$

The confidence interval CI in the native parameter representation is then given by inverse-transforming each of these values in turn:

$$CI = \exp(CI')/(1 + \exp(CI)). \quad (67)$$

(using $CI = \exp(CI')$ for the three σ-parameters). Confidence intervals calculated in this manner for the four model parameters are reported in the main text.

Proportion of Genetically-determined Phenotypic Variance Explained

With a slight abuse of notation, denoted the contribution to phenotypic variance from SNP j as $\text{var}(Y|g_j)$. From Eq. 1 in the univariate setting, and assuming Hardy-Weinberg equilibrium, the contribution $\text{var}(Y|g_j)$ under an additive polygenic model is

$$\text{var}(Y|g_j) = \text{var}(\beta_j g_j) = 2\beta_j^2 p_j(1 - p_j). \quad (68)$$

From Eqs. 13, and 14, the estimated $\hat{\beta}$ incorporates the true effect size and a residual component. The contribution of the SNP to phenotypic variance arises only from the genetic effect component, i.e., from δ not $\delta + \epsilon$. Using Eq. 14, it can be seen that

$$\text{var}(Y|g_j) \propto \delta_j^2. \quad (69)$$

From this it follows that the fraction of the total variance in genetic liability for SNPs j exceeding a z-score threshold z_t can be estimated from points given by

$$\frac{\sum_{j:|z_j|>z_t} E(\delta^2|z_j)}{\sum_{all\ j} E(\delta^2|z_j)}, \quad (70)$$

where $E(\delta^2|z_j)$ is given by Eq. 59. The expected fraction of the total variance in genetic liability can then be estimated by summing over equally-spaced z-score bins (indexed by k) weighted by the z-score PDF (Eq. 37):

$$tv(z_t) = \frac{\sum_{k:|z_k|>|z_t|} f(z_k)E(\delta^2|z_k)}{\sum_{all\ k} f(z_k)E(\delta^2|z_k)}. \quad (71)$$

Note that from Eqs. 57 and 59, the posterior expectation due to sparse effects is given by the product $tdr(z)E_s(\delta^2|z)$, where

$$E_s(\delta^2|z) = \mu_s^2(z) + \sigma_s^2(z). \quad (72)$$

Therefore, if only the sparse effects are assumed to contribute to phenotypic variance, Eq. 71 becomes:

$$tv(z_t) = \frac{\sum_{k:|z_k|>|z_t|} f(z_k)tdr(z)E_s(\delta^2|z_k)}{\sum_{all\ k} f(z_k)tdr(z)E_s(\delta^2|z_k)}. \quad (73)$$

In integral form,

$$tv(z_t) = \frac{\int_{z:|z|>z_t} f(z)tdr(z)E_s(\delta^2|z)dz}{\int_{-\infty}^{\infty} f(z)tdr(z)E_s(\delta^2|z)dz}. \quad (74)$$

Multistage Design Combining Independent Discovery and Replication Datasets

Eq. 49 gives the probability $Pr(z_r|z_d)$ of obtaining z_r for a SNP in a replication dataset, of sample size N_r, given z_d for the SNP in an independent discovery dataset, of sample size N_d. Now consider combining both datasets, with $N_{dr} = N_d + N_r$. What is the probability of z_d replicating in the combined dataset? Given z_d in the discovery dataset, if one obtains z_r in the independent replication dataset, then in the combined dataset one has $z_{dr} = w_d z_d + w_r z_r$, where $w_d = \sqrt{N_d/N_{dr}}$ and $w_r = \sqrt{N_r/N_{dr}}$. Since z_d is fixed (not a random variable), one has $Pr(z_{dr}|z_d) = Pr(z_r|z_d)$ (in this simplified notation, the left-hand side is the posterior probability of obtaining z_{dr} in the combined data set, while the right-hand side is the posterior probability of obtaining z_r in the independent replication portion). Define replication such that the z-score in the replication dataset passes a chosen threshold z_t, e.g., $z_t \simeq -5.33$ corresponding to $p_t = 5 \times 10^{-8}$. Then the probability of z_d replicating in the full dataset is the probability that $-|z_{dr}| < z_t$, which is the probability that

$$-|z_r| < \frac{z_t + w_d|z_d|}{w_r} \equiv z'_t(z_t, z_d). \quad (75)$$

Since the distribution of $z_r|z_d$ for the complementary replication subset is given by Eq. 49, the probability of z_d replicating in the complementary replication dataset is the probability of getting $-|z_r| < z_t$, which is equal to the proportion of z-scores satisfying that inequality, which is simply given by the corresponding CDF with threshold z_t (see Eq. (21) in the main paper). Then, for replication in the combined dataset, all that has changed is that there is a new threshold $z'_t(z_t, z_d)$ for z_r to pass. So for the combined dataset, the replication rate is

$$R_{dr}(z_d; z_t) = (1 - tdr)\Phi(z'_t, -|m_u|, s_u^2) + tdr \cdot \Phi(z'_t, -|m_s|, s_s^2) \quad (76)$$
$$\equiv Pr(p_{dr} < p_t) \quad (77)$$

where p_{dr} is the p-value for the SNP in the combined data set.

References

de Bakker, P. I., Ferreira, M. A., Jia, X., Neale, B. M., Raychaudhuri, S., Voight, B. F., 2008. Practical aspects of imputation-driven meta-analysis of genome-wide association studies. Human molecular genetics 17 (R2), R122–R128.

Devlin, B., Roeder, K., Dec 1999. Genomic control for association studies. Biometrics 55 (4), 997–1004.

Efron, B., 2013. Large-scale inference : empirical Bayes methods for estimation, testing, and prediction. Cambridge University Press, Cambridge, UK New York.

Lee, S. H., Goddard, M. E., Wray, N. R., Visscher, P. M., 2012. A better coefficient of determination for genetic profile analysis. Genetic epidemiology 36 (3), 214–224.

Liptak, T., 1958. On the combination of independent tests. Magyar Tud Akad Mat Kutato Int Kozl 3, 171–197.

Mosteller, F., Bush, R. R., 1954. Selected quantitative techniques. In: Lindzey, G. (Ed.), Handbook of Social Psychology. Vol. 1. Addison-Wesley, Cambridge, Mass., pp. 289–334.

Robinson, G. K., 1991. That blup is a good thing: the estimation of random effects. Statistical science, 15–32.

Wellek, S., Ziegler, A., 2012. Cochran-armitage test versus logistic regression in the analysis of genetic association studies. Human heredity 73 (1), 14–17.

Zaykin, D. V., 2011. Optimally weighted z-test is a powerful method for combining probabilities in meta-analysis. Journal of evolutionary biology 24 (8), 1836–1841.

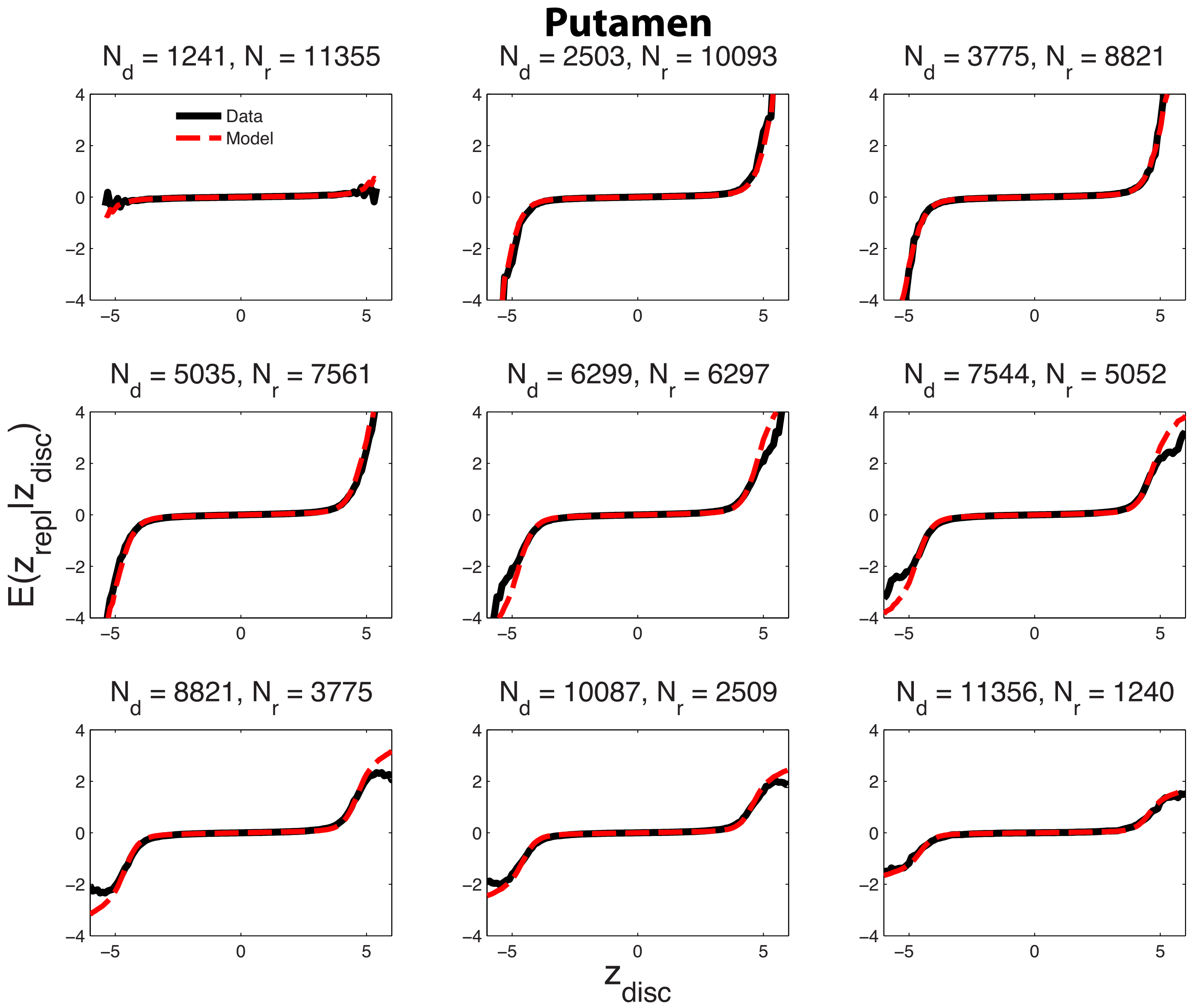
Putamen
N_d = 1241, N_r = 11355
Data
Model
N_d = 2503, N_r = 10093
N_d = 3775, N_r = 8821
N_d = 5035, N_r = 7561
N_d = 6299, N_r = 6297
N_d = 7544, N_r = 5052
N_d = 8821, N_r = 3775
N_d = 10087, N_r = 2509
N_d = 11356, N_r = 1240
E(z_repl|z_disc)
z_disc

Figure 4:

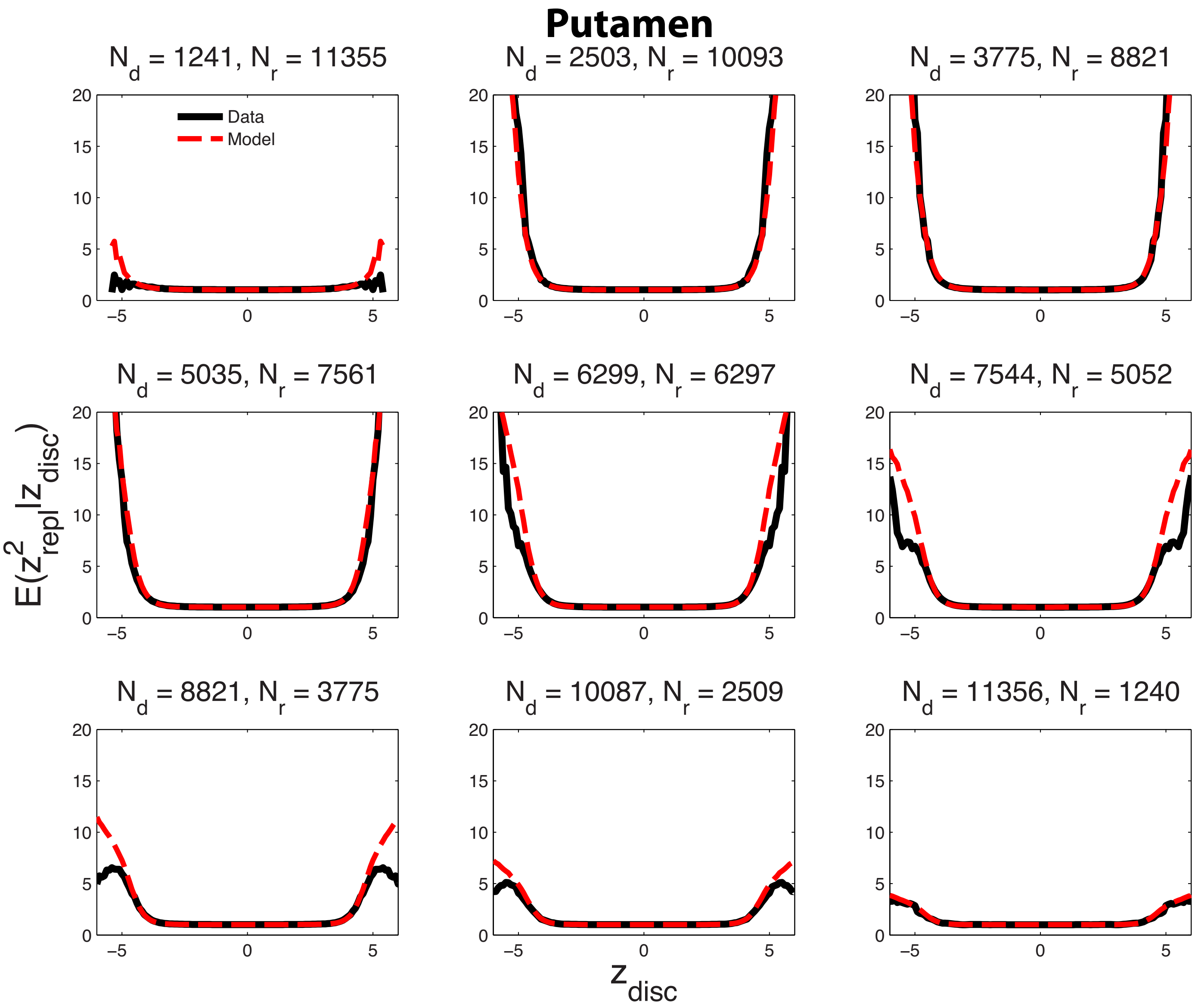
Putamen
N_d = 1241, N_r = 11355
Data
Model
N_d = 2503, N_r = 10093
N_d = 3775, N_r = 8821
N_d = 5035, N_r = 7561
N_d = 6299, N_r = 6297
N_d = 7544, N_r = 5052
N_d = 8821, N_r = 3775
N_d = 10087, N_r = 2509
N_d = 11356, N_r = 1240
E(z^2_repl|z_disc)
z_disc

Figure 5:

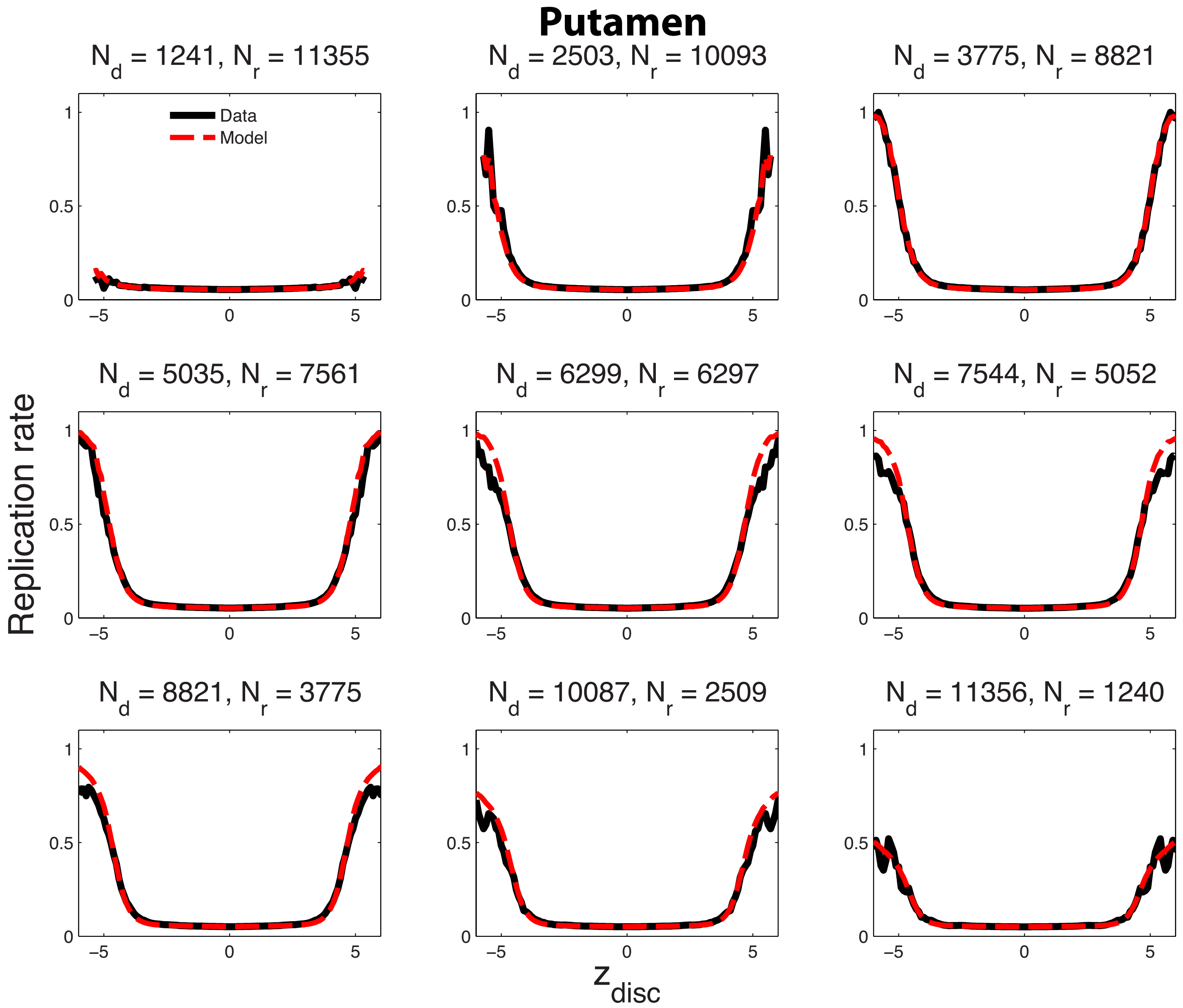
Putamen
N_d = 1241, N_r = 11355
Data
Model
N_d = 2503, N_r = 10093
N_d = 3775, N_r = 8821
N_d = 5035, N_r = 7561
N_d = 6299, N_r = 6297
N_d = 7544, N_r = 5052
N_d = 8821, N_r = 3775
N_d = 10087, N_r = 2509
N_d = 11356, N_r = 1240
Replication rate
z_disc

Figure 6:

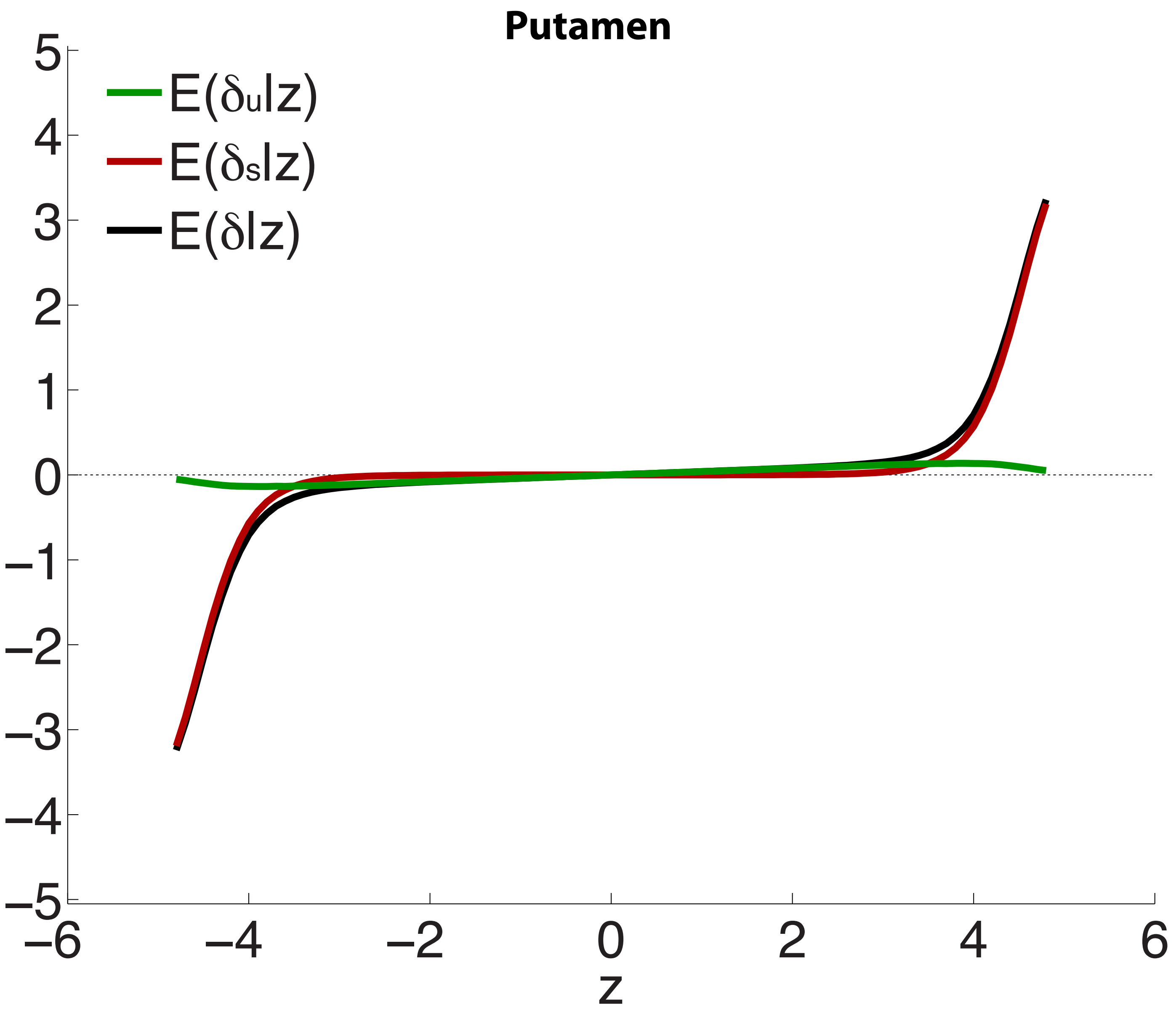

Figure 7:
